# The role of characterisation in everyday voice engagement and AVATAR therapy dialogue

**DOI:** 10.1017/S0033291721000659

**Published:** 2022-12

**Authors:** Thomas Ward, Rachel Lister, Miriam Fornells-Ambrojo, Mar Rus-Calafell, Clementine J. Edwards, Conan O'Brien, Tom KJ Craig, Philippa Garety

**Affiliations:** 1 Department of Psychology, Institute of Psychiatry, Psychology & Neuroscience, King's College London, London, UK; 2 South London & Maudsley NHS Foundation Trust, London, UK; 3 Research Department of Clinical, Educational and Health Psychology, University College London, London, UK; 4 Mental Health Research and Treatment Center, Faculty of Psychology, Ruhr-Universität Bochum, Bochum, Germany; 5 Department of Health Service and Population Research, Institute of Psychiatry, Psychology & Neuroscience, King's College London, London, UK

**Keywords:** Auditory hallucinations, digital health, personification, relational therapy, voices

## Abstract

**Background:**

Voices are commonly experienced as communication with a personified ‘other’ with ascribed attitudes, intentionality and personality (their own ‘character’). Phenomenological work exploring voice characterisation informs a new wave of relational therapies. To date, no study has investigated the role of characterisation in behavioural engagement with voices or within psychological therapy for distressing voices.

**Methods:**

Baseline characterisation (the degree to which the voice is an identifiable and characterful entity) of the dominant voice was rated (high, medium or low) using a newly developed coding framework, for *n* = 60 people prior to starting AVATAR therapy. Associations between degree of characterisation and (i) everyday behavioural engagement with voices (The Beliefs about Voices Questionnaire-Revised; *n* = 60); and (ii) interaction within avatar dialogue [Session 4 Time in Conversation (participant–avatar); *n* = 45 therapy completers] were explored.

**Results:**

Thirty-three per cent reported high voice characterisation, 42% medium and 25% low. There was a significant association between characterisation and behavioural engagement [*H*(2) = 7.65, *p* = 0.022, *ɛ*2 = 0.130] and duration of participant–avatar conversation [*F*(2,42) = 6.483, *p* = 0.004, *η*2 = 0.236]. High characterisation was associated with increased behavioural engagement compared with medium (*p* = 0.004, *r* = 0.34; moderate effect) and low (*p* = 0.027, *r* = 0.25; small−moderate effect) with a similar pattern observed for the avatar dialogue [high *v.* medium: *p* = 0.008, Hedges’ *g* = 1.02 (large effect); high *v.* low: *p* = 0.023, Hedges' *g* = 1.03 (large effect)]. No differences were observed between medium and low characterisation.

**Discussion:**

Complex voice characterisation is associated with how individuals interact with their voice(s) in and out of therapy. Clinical implications and future directions for AVATAR therapy and other relational therapies are discussed.

## Introduction

The way I always see it is, he reminds me of a tradesman that's done some kind of office work for 30 years… And he's got a lot of…stories to tell you about it. …. he's lived the lifestyle that…that he'd be happy with. He knows […] I need a proper conversation with him to find direction in life and he ignores that. [John (35)]

Influential models of auditory verbal hallucinations (henceforth voices) outline how internally generated stimuli (often related to inner speech and/or memory) come to be ‘misattributed’ to an external source as a result of biopsychosocial factors including difficulties in self- and source-monitoring (David, 2004; Waters et al., 2012). Some authors have proposed moving away from an individualistic focus on ‘aberrant’ auditory perception to consider voices as experiences of perceived communication with an ‘other’ (Deamer & Wilkinson, 2015; Wilkinson & Bell, 2016). This includes growing interest in the role of broader social-cognitive processes to explain why voices, like John's, are not simply ‘alien’ to the self, but experienced as specific, characterised social agents (Alderson-Day & Fernyhough, 2016; Bell, 2013; Wilkinson & Bell, 2016).

According to phenomenological studies, around 70% of voices are reported as ‘characterful’ (Woods, Jones, Alderson-Day, Callard, & Fernyhough, 2015), i.e. associated with specific character traits, including experiences, attitudes, animacy and beliefs. A recent study (Alderson-Day et al., 2021) found evidence of ‘complex personification’ of voices in a significant minority (40%) of a sample of people attending early intervention for psychosis services (*n* = 60). While voice severity scores were very similar in groups with and without complex personification, there was an association between complex personification and experiencing voices as conversational and companionable (Alderson-Day et al., 2021). Beavan (2011) identifies the experience of forming a relationship with a ‘characterised’ identity as an essential aspect of the experience, and proposes that voices can be important signifiers of a person's life history and relationship to their (social) world (see also Birchwood et al., 2004; Corstens, Longden, McCarthy-Jones, Waddingham, & Thomas, 2014; Hayward, Berry, & Ashton, 2011; Longden, Corstens, Escher, & Romme, 2012; Romme, Escher, Dillon, Corstens, & Morris, 2009).

This reframing of what it means to ‘hear a voice’ brings potentially significant implications for psychological approaches to distressing voices. Birchwood and colleagues (Birchwood et al., 2004; Birchwood, Meaden, Trower, Gilbert, & Plaistow, 2000) have integrated their seminal cognitive model (Birchwood & Chadwick, 1997; Chadwick & Birchwood, 1994) with a ‘social mentalities’ approach which posits that humans have evolved mechanisms for recognising dominant–subordinate interactions, i.e. their social rank (Gilbert et al., 2001; Gilbert & Allan, 1994). Early experiences of powerlessness and inferiority within social relationships may establish social schemata that drive the subsequent appraisals of voices, and ultimately lead to significant levels of distress and depression (Birchwood et al., 2004). Systematic reviews support the view that social schemata may mediate the cognitive appraisal–distress relationship with the implication that therapies could benefit from targeting social and interpersonal variables (Mawson, Cohen, & Berry, 2010; Paulik, 2012). These theoretical developments have informed a specific cognitive therapy for command hallucinations, which in a randomised controlled trial (Birchwood et al., 2014) reported significant changes in specific treatment targets (namely compliance behaviours and the power difference between voice-hearer and voice).

More recently, a new wave of relational approaches has emerged which focuses on the interpersonal relationship between the hearer and voice [see e.g. Talking with Voices and Making sense of voices (Corstens, Longden, & May, 2012; Steel et al., 2019)]; Relating Therapy (Hayward, Jones, Bogen-Johnston, Thomas, & Strauss, 2017; Hayward, Overton, Dorey, & Denney, 2009); AVATAR therapy (Craig et al., 2018; Leff, Williams, Huckvale, Arbuthnot, & Leff, 2013; Leff, Williams, Huckvale, Arbuthnot, & Leff, 2014; Ward et al., 2020). A key aspect of these relational approaches is the representation of the social agent to which the voice is ascribed (Deamer & Wilkinson, 2015) with voice personification and characterisation brought to the fore. AVATAR therapy involves real-time ‘face-to-face’ dialogue between the person and a computerised representation of their main (dominant) persecutory voice (enacted by the therapist using voice transformation software). The typical AVATAR therapy structure involves an initial focus on the person asserting themselves over verbatim voice content, delivered by the avatar to mirror the daily voice experience (Session 1–3). Time spent in direct dialogue in early sessions tends to be short (~5 min) and is influenced by individual differences in anxiety and acclimatisation to the set-up. Over time the avatar transitions to a more conciliatory position cueing a focus on self-esteem, developing a shared understanding of the voice and working towards the person reclaiming power and control in their life (Sessions 4–6). From Session 4 onwards the dialogue tends to become more extended as the therapy targets a realistic enactment of the ascribed character and background of the voice reflecting the person's beliefs (e.g. identity, power, intention and the consequences of resistance (Chadwick & Birchwood, 1994) and how they experience voice characterisation when relating to their voice. The ultimate aim of AVATAR therapy is for the person to experience increased power, control and confidence within the avatar dialogue which is generalised to the daily voices and other social relationships (see also Hayward et al., 2017; Steel et al., 2019). While voice characterisation has been considered likely to impact on AVATAR therapy delivery (Craig, Rus-Calafell, & Ward, 2016; Ward et al., 2020) its specific influence on therapy remains an open question. Indeed while ‘characterful’ voices appear common among voice-hearers (Beavan, 2011; Woods et al., 2015), attempts to understand associations between voice characterisation and other variables are only just emerging and, to date, no empirical study has investigated its role in psychological therapy for voices.

### Aims

This study aims to explore whether the extent of everyday voice engagement and participant–avatar interaction within AVATAR therapy are associated with the degree to which the person's dominant voice is experienced as a highly characterised social agent.

### Hypotheses

1. Higher voice characterisation will be associated with increased behavioural engagement with voices.
2. Higher voice characterisation will be associated with increased interaction (longer time in direct dialogue) with a digital representation of the voice (‘the avatar’) within AVATAR therapy.

### Methods

This study tests associations between voice characterisation (coded high, medium or low from pre-therapy assessments) and (i) a standardised measure of behavioural engagement [The Beliefs about Voices Questionnaire-Revised (BAVQ-R); assessed pre-therapy] and (ii) time spent in direct dialogue with ‘avatar’ (digital representation of voice) during Session 4 of AVATAR therapy intervention.

### Participants

Sixty participants allocated to receive AVATAR therapy were randomly selected from a larger clinical trial of AVATAR therapy [approved by the London-Hampstead Research Ethics Committee (Reference 13/Lo/0482) (Craig et al., 2018)]. The inclusion criteria: aged over 18 years, ability to speak and read English, and have experienced troubling auditory hallucinations for at least 12 months. Exclusion criteria: aged under 18 years, unable to give informed consent, currently in receipt of cognitive behaviour therapy for psychosis, refusing all medication, a diagnosis of organic brain disease or a primary substance dependency or auditory hallucinations in a language not spoken by the therapists. A total of 45 participants engaged with therapy such that a recorded Session 4 was available and therefore this subset was used to test Hypothesis 2.

### Measures

*Characterisation:* A novel phenomenological coding framework was developed to derive a measure of the degree of characterisation of the person's main voice. Characterisation reflected the degree to which the voice was experienced as an identifiable and characterful entity. Informed by previous phenomenological work (see e.g. Woods et al., 2015), the coding framework took into account identified physical characteristics, identity and psychosocial characteristics. Presence of more of these features and aspects carrying greater complexity/detail indicated higher degree of characterisation. Three levels (high, medium or low; see Table 3) were defined with the rationale that the degree of characterisation might be particularly relevant to the AVATAR therapy context. A clear identity (whether the person was explicitly ‘known’ in the sense of a ‘real-life’ referent or an identifiable stranger) was a requirement for a coding of high characterisation, and an exclusion for coding of low characterisation. The framework was used to code transcripts of the baseline assessment on the AVATAR trial as well as the clinical interview which precedes the commencement of AVATAR therapy [this clinical assessment included the question ‘Does it feel as though the voice(s) that you hear have their own character or personality?’]. The focus of these assessments was on the main/dominant voice as this is represented by the created ‘avatar’ and targeted within AVATAR therapy. Following the initial development of the coding frame, five participants were randomly selected for pilot coding by authors (RL and TW). Through this process the framework was refined to ensure reliability and accurate reflection of the data. RL then coded the remaining sample with 10 participants randomly selected and independently coded by TW; interrater reliability for the characterisation variable was 0.88 (quadratic weighting). There was 100% agreement for ratings involving high characterisation; three disagreements in ratings of low and medium categories were resolved by further discussion.

*BAVQ-R* (Chadwick, Lees, & Birchwood, 2000): a validated self-report measure of a person's appraisals of their voices, including perceived malevolence, benevolence and omnipotence (six items each), and styles of relating to the voice; behavioural engagement, emotional engagement, emotional resistance (four items each), and behavioural resistance (five items). Items are scored from 0 (disagree) to 3 (strongly agree). The scale has good validity and good reliability (e.g. Cronbach's alpha for engagement of 0.87 and for resistance of 0.85) (Chadwick et al., 2000). Although the BAVQ-R response scales have been validated using a total including both emotional and behavioural items, subscales have been reported separately (Birchwood et al., 2014). Behavioural engagement subscale score was used in the current study as a measure of active engagement with the voice (e.g. items: When I hear my voice usually: ‘I seek the advice of my voice’; ‘I willingly follow what my voice tells me to do’; ‘I listen to it because I want to’; ‘I have done things to start to get in contact with my voice’). Additional response subscales were included in exploratory analysis: emotional engagement (‘My voice reassures me/makes me happy/makes me feel calm/makes me feel confident’); behavioural resistance (e.g. ‘When I hear my voice I usually tell it to leave me alone/try to stop it’); emotional resistance (e.g. “My voice frightens me/makes me feel down).

*Interaction with ‘avatar’ within AVATAR therapy dialogue:* Session 4 recordings (*n* = 45) were transcribed and used to measure engagement in dialogue with the avatar. The primary measure of interaction was total conversation time (TCT), between the person and the avatar, in seconds. Secondary variables were: total number of words (TNW) spoken by person and avatar during the conversation, number of exchanges (NoE) between avatar and person and number of words spoken by the person (NWP). TCT was calculated using the timer displayed on Windows Media Player and ‘number of words’ data were calculated using the ‘word count’ function in Microsoft Word. Time or words captured by these measures excluded any time spent talking with the therapist. Session 4 was selected as most reflective of elaborated dialogue given the typical AVATAR therapy structure. Example Session 4 dialogue: Avatar: ‘You seem different, I can't hold you down like before, what is changing?’; person: ‘I'm not that little girl anymore, I'm a grown woman, you will not have this power over me anymore’.

## Analysis

Analysis was conducted using Stata Version 16. Behavioural engagement (BAVQ-R) showed significant positive skew which could not be corrected using log transformation, therefore the non-parametric Kruskal–Wallis test was used to analyse associations between characterisation and behavioural engagement, with pairwise post-hoc Dunn tests to compare levels (high, medium and low). Univariate analysis of variance, with post-hoc Tukey testing, was used to analyse differences in TCT during AVATAR therapy dialogue for the different levels of voice characterisation (high, medium and low). To increase the power given the relatively small group sizes, secondary analyses were conducted with groups dichotomised as high (complex) characterisation *v.* medium/low (non-complex) characterisation (see Alderson-Day et al. (2021) for a comparable approach) – Mann–Whitney *U* tests were used for Hypothesis 1 and independent *t* tests for Hypothesis 2.

## Results

Data from 60 participants were included in this study; ages ranged between 19 and 67 years old (to the nearest year), with a mean age of 42 years. Table 1 shows demographics, clinical characteristics and engagement variables.

**Table 1.** Demographics for the sample (*n* = 60)

	*n*	%
Gender		
Female	15	25.0
Male	45	75.0
Ethnicity		
White British	23	38.3
Black British	8	13.3
Black Caribbean	5	8.3
Black African	7	11.7
South Asian	2	3.3
Other	15	25.0
Level of education		
Primary	13	21.7
Secondary or a level equivalent	21	35.0
Vocational education	13	21.7
University degree/professional qualif.	13	21.7
Diagnosis		
Paranoid schizophrenia	42	70.0
Schizoaffective disorder	8	13.3
Bipolar disorder	1	1.7
Unspecified non-organic psychosis	5	8.3
Depression with psychotic symptoms	4	6.7
Age of onset of illness		
8–10	4	6.7
11–20	26	43.3
21–30	24	40.0
31–60	6	10.0
Marital status		
Married/cohabiting	2	3.3
In a steady relationship	3	5.0
In a casual relationship	3	5.0
Single	44	73.3
Divorced/separated	7	11.7
Widowed	1	1.7
	Mean (s.d.)	s.d.
BAVQ-R (responses)		
Behaviour engagement (possible range 0–12)	2.10	2.47
Emotional engagement (possible range 0–12)	1.53	2.53
Engagement total (possible range 0–24)	3.63	4.63
Behavioural resistance (possible range 0–15)	9.85	3.56
Emotional resistance (possible range 0–12)	8.98	2.54
Resistance total (possible range 0–27)	18.82	4.86
Avatar dialogue (*n* = 45) (time in seconds)		
Total conversation time (TCT)	742.42	293.94
Total number of words spoken	1629.53	756.83
Number of words spoken by person (NWP)	869.60	576.46
Number of words spoken by avatar (NWA)	759.93	345.12
Number of exchanges (NoE)	115.29	58.81

### Descriptive data on voices

Although most participants reported multiple voices (82%, *n* = 49), assessments reported in the current paper were anchored to the dominant voice, as this was used to create the avatar. The majority of dominant voices were male with ‘stranger’ the most common identity category (see Table 2).

**Table 2.** Ascribed gender and identity of the main voice

	*n*	%
Gender		
Male	39	65.0
Female	11	18.3
Both	3	5.0
Unknown	6	10.0
Missing	1	1.7
Identity		
Family	4	6.7
Friends	6	10.0
Employer/colleague	1	1.7
Strangers	40	66.7
Non-humans	2	3.3
Deity/religious being	2	3.3
Neighbours	2	3.3
Public figure	2	3.3
Unknown	1	1.7

All participants heard intelligible voice content, but almost half (45%, *n* = 27) also heard vague or unintelligible content. For almost all participants (95%, *n* = 57), voices addressed the participant in the second person at least some of the time, however, 42% (*n* = 25) of people had voices using a mixture of forms of address. All participants reported at least some of their voice content as communicative – this included 100% reporting at least one verbatim voice comment that had a literal meaning (typically relating to criticism, abuse and threat), as well as over half reporting a non-literal ‘communicative intent’ (57%, *n* = 34), e.g. the voice drawing the attention to a chocolate bar wrapper on the floor taken to mean ‘it's there to say eat lots of chocolate and get fat’; a voice command ‘go sit outside’ taken to mean the voice coercing the person to become homeless. The degree of characterisation of the voice (three categories: high, medium and low), with frequencies, defining features and examples, are shown in Table 3.

Hypothesis 1: higher voice characterisation will be associated with increased behavioural engagement with voices

**Table 3.** Degree of characterisation of voices (high, medium and low) with associated typical features and examples

Characterisation	*n*	%	Definition and typical features	Example
High	20	33	Complex characterisation. A clear identity (whether known or unknown), including evidence of: - ascribed background, e.g. age, gender, sound qualities of the voice, presence of an accent, indications of class, accompanying image or visual description of the voice, ascribed autobiography. - idiosyncratic personality/character, e.g. attitudes, preferences, characteristic actions/behaviours, specific likes/dislikes/preferences, ascribed thoughts and complex intentions.	Known: A client's deceased father; the voice related to the client in the same way as the father did while alive, e.g. calling him a ‘wimp’ due to a specific set of beliefs the voice of the father held and its own insecurities. Ascribed intentions, personality, characterised actions and experiences (mirroring autobiographical relationship) ‘he keeps me safe and unsafe, in some ways he's caring…[but] he ruined my life…always nagging in my head’. Unknown: John's vividly characterised stranger: ‘A tradesman who has lived a lifestyle’ (see Introduction).
Medium	25	42	Voices experienced with ‘one-dimensional’ characterisation or stereotypical person-like presentations [e.g. an angry man, an old woman (Woods et al., 2015)], or archetypal characters (e.g. the devil, the trickster, the hero, the god/goddess). Spiritual entities with some (basic) anthropomorphic traits.	A voice described as an ‘evil jealous spirit’, ‘linked to the devil’; the voice had an accent, recurrent identity and deliberate actions for example it ‘plays’ on the client's tinnitus and ‘it makes trouble and then it runs away…inciting a riot’. Rated as medium not high due to lacking complexity in ascribed character/thought/action (the voice was essentially an archetypal ‘malevolent trickster’).
Low	15	25	Anonymous or ‘incognito’ voices (Hayward, 2003). No evidence of clear identity or individual personality/character. Only identified by broad gender and speech content with no specific character attributes identified. Intentions do not clearly extend beyond the verbatim content, e.g. content: ‘kill yourself’ and intention: voice wants me to come to harm/die.	Male voice that swore at the hearer saying that they want to kill the person and that they were ‘no good’. Voice described simply in terms of being ‘scary and aggressive’. The voice was ascribed no specific identity, beliefs, thoughts or idiosyncratic intentions.

There was a significant association between degree of voice characterisation and behavioural engagement (BAVQ-R) as shown in Table 4 (moderate effect size). As hypothesised, those with highly characterised voices showed higher behavioural engagement when compared with those with medium characterisation (moderate effect size) and low (small-moderate effect size). There were no significant differences in behavioural engagement between low and medium characterisation. Exploratory analysis was conducted for the other response scales of BAVQ-R. There was a significant association between characterisation and emotional engagement (moderate effect size). Those with highly characterised voices showed significantly higher emotional engagement when compared with medium (moderate effect size) and low (small-moderate effect size) characterisation. There were no associations between voice characterisation and resistance (behavioural or emotional).

Hypothesis 2: higher voice characterisation will be associated with increased interaction with a digital representation of the voice ('the avatar’) within AVATAR therapy

**Table 4.** Engagement and resistance (behavioural and emotional) across different levels of voice characterisation

	High (*n* = 20)	Medium (*n* = 25)	Low (*n* = 15)	Test statistics
Behavioural engagement: median (IQR); mean rank	3(3.5); 38.83	0(3), 25.46	0(4); 27.80	*H*(2) = 7.65, *p* = 0.022, *ɛ*^2^ = 0.130 High *v.* Med: *z* = 2.670, *p* = 0.004, *r* = 0.34 High *v.* Low: *z* = 1.934, *p* = 0.027, *r* = 0.25 Med *v.* Low: *z* = −0.429, *p* = 0.334, *r* = 0.06
Exploratory analysis				
Emotional engagement: median (IQR); mean rank	2(5); 38.60	0 (0); 26.16	0(1); 26.93	*H*(2) = 8.469, *p* = 0.015, *ɛ*^2^ = 0.144 High *v.* Med: *z* = 2.716, *p* = 0.003, *r* = 0.35 High *v.* Low: *z* = 2.237, *p* = 0.013, *r* = 0.29 Med *v.* Low: *z* = −0.155, *p* = 0.438, *r* = 0.02
Behavioural resistance: median (IQR); mean rank	10 (7.5); 28.50	10 (5); 32.10	10 (6); 30.50	*H*(2) = 0.477, *p* = 0.788, *ɛ*^2^ = 0.008
Emotional resistance median (IQR); mean rank	8.5 (3.5); 28.83	9 (3); 29.06	9 (4); 35.13	*H*(2) = 1.457, *p* = 0.483, *ɛ*^2^ = 0.025

*Note:* For non-parametric tests effect size *r* = *z*/√*N*, where *z* = standardised test statistic. *N* = number of observations (Rosenthal, 1991); 0.1 = small, 0.3 = medium, 0.5 = large.

The mean TCT across the sample (*n* = 45) was 742.42 s (s.d. = 293.94). There was a significant effect of the degree of characterisation on TCT (Table 5). Post-hoc tests indicated that TCT was significantly greater for those with high characterisation, than those with low or medium characterisation (large effect sizes); no significant difference was found between low and medium characterisation (Table 5).

**Table 5.** Total duration of active dialogue between person and avatar (during Session 4)

	High character (*N* = 18)	Medium character (*N* = 18)	Low character (*N* = 9)	Test statistics
TCT mean (s.d.)-secs	915.11 (303.32)	633.61 (231.91)	614.67 (230.88)	F (2,42) = 6.483, *p* = 0.004, *η*^2^ = 0.236
Post-hoc	Mean difference (95% CI)	2-tailed *p* (Tukey)	Hedges' *g* (95% CI)	
High *v.* low	300.44 (32.72 to 568.17)	0.021	1.03 (0.20 to 1.85)	
High *v.* medium	281.5 (62.90 to 500.10)	0.007	1.02 (0.33 to 1.70)	
Medium *v.* low	18.94 (−248.78 to 286.67)	0.983	0.08 (−0.70 to 0.85)	

*Note*: TCT = total conversation time (seconds).

As a sensitivity check, three other objective measurements of engagement with the avatar (TNW spoken, NWP, NoE) were tested for associations with voice characterisation. While the ‘TNW’ and ‘NWP’ variables showed the same pattern reported above, the ‘NoE’ variable did not show a significant association with characterisation (see Online Supplementary Table S1).

### Secondary analyses

Secondary analyses comparing high (complex) with low/medium (non-complex) characterisation showed significant differences in behavioural and emotional engagement (moderate effect sizes) but not resistance scales (see Online Supplementary Table S2). Avatar dialogue interaction was significantly greater in those with high (complex) characterisation than low/medium (non-complex) for all measures – effect sizes were large except for NoE which showed moderate effect sizes (see Online Supplementary Table S3).

## Discussion

This study builds on the conceptualisation of voices as social experiences of communication with an ascribed ‘other’. Voice characterisation has been investigated with respect to everyday voice interaction and, for the first time, engagement within AVATAR therapy for distressing voices. The findings suggest that more highly characterised dominant voices (a third of the sample) were associated with increased behavioural engagement with voices which was mirrored in greater interaction during AVATAR therapy dialogue.

The findings that just over 70% of the sample reported some degree of voice characterisation (i.e. high or medium) is broadly consistent with other phenomenological research in this area (Beavan, 2011; Woods et al., 2015), suggesting that voices are ‘characterful’ to some extent for the majority of voice-hearers. While the most common voice identity was ‘unknown/strangers’, all reported communicative intent from their voice (literal and in some cases also non-literal). Similarly, Alderson-Day et al. (2021) found (within a sample attending an early intervention service) that 75% of voices recurred over time, had a distinct character, but were not related to a known person [i.e. experienced as an ‘internally individuated agency’ (Wilkinson & Bell, 2016)]. The key differences observed in the current study related specifically to highly characterised voices compared with medium (one-dimensional characterisation) or low (amorphous voices); no consistent differences were observed between low and medium characterisation.

There is increasing interest in how, when and why voices become personified and whether characterisation evolves over time. Personification may emerge early for some [where social cognitive and neurocognitive processes are primed for the ascription of agency (Bell, 2013)], while for others voices may remain essentially non-personified experiences. Previous findings of complex personification at the point of first clinical contact and absence of association with time since onset suggest that personification cannot be easily reduced to a secondary consequence of beliefs about voices (Alderson-Day et al., 2021). The study of Alderson-Day et al. (2021) also reported that complex personification (significantly overlapping in definition with ‘high characterisation’ in the current study) was found in 40% of participants and associated with experiencing voices as conversational and companionable. This is consistent with the finding in the current study of an association between high characterisation (33% of the current sample) and behavioural engagement (see also John's ‘need for a conversation’ with his highly characterised voice in the opening quote). It should be noted that voices inhabit a complex phenomenological and developmental landscape in which establishing primacy and reciprocal influence between beliefs, experiences and personification is challenging. Interpersonal experiences (including current discrimination or conversely positive new relationships) evolve over time, continuing to shape experiences in ways that may be missed in cross-sectional studies (see also Longden et al., 2012).

Despite the findings in support of the main study hypotheses, important questions remain. Overall ratings of behavioural engagement (BAVQ-R) were notably low. This is unsurprising in a sample recruited for recurrent distressing voices, given the association of BAVQ-R engagement with benevolent voices (Chadwick et al., 2000). There are also important clinical questions as to how ‘voice engagement’ might vary across individuals and contexts. Higher levels of engagement may reflect active dialogue with positive aspects of voices, consistent with the similar pattern of findings found for positive emotional engagement (albeit again with low overall levels). The interpersonal dimension of ‘proximity’, with opposing poles of ‘withdrawal/self-isolation’ and ‘over-involvement/intrusiveness’ (Hayward et al., 2011) has been identified as important in the context of distressing voices (Birtchnell, 1996; Hayward et al., 2011). This underscores the diversity of voices and the role of context (current and autobiographical) when considering relating to voices (Hayward et al., 2017). Qualitative research has started to explore relational change in voices over time (Bogen-Johnston, deVisser, Strauss, & Hayward, 2019; Hartigan, McCarthy-Jones, & Hayward, 2014). Future longitudinal studies exploring the potential evolution of voice characterisation over time would be of significant theoretical and clinical importance. Experience-sampling methodology (Myin-Germeys et al., 2018) may also illuminate further the ways in which characterisation intersects with voice engagement, distress and daily activity and how this might evolve (e.g. before and after therapy).

This study provides the first empirical evidence that baseline voice characterisation predicts person–avatar interaction during active therapy dialogue. TCT was selected as the best proxy for active engagement, with the rationale that extended dialogue increases the opportunity to address treatment targets including work on past relational conflicts which can be mirrored in the voice (Ward et al., 2020). However, linguistic analysis of dialogic exchanges might identify more nuanced aspects of the therapy process especially relating to enacted characterisation and the ‘pragmatic’ opportunities within dialogue (Deamer & Wilkinson, 2015).

Clearly a crucial question is whether the influence of voice characterisation on engagement in AVATAR dialogue leads to improvement in the actual distressing voices. Rus-Calafell et al. (2020) have demonstrated that the interaction between sense of voice presence (i.e. virtual embodiment of the ‘voice as avatar’) and reduction of anxiety predicted improvement in two of the significant AVATAR therapy outcomes: PSYRATS (Haddock, McCarron, Tarrier, & Faragher, 1999) total and frequency of voices. Future research could investigate whether voice characterisation influences sense of presence and has an impact on treatment outcome and we plan to investigate this (we discuss in more detail below, in clinical implications, our planned exploration of characterisation as a potential moderator of treatment outcome in the new AVATAR2 clinical trial).

### Limitations

This study reports associations and there is a possibility of unmeasured causes in addition to uncertainty concerning the direction of causation. Future longitudinal and interventional studies may elucidate these relationships. The recruitment of the sample with distressing voices means that findings may not generalise to people who experience voices without associated distress. Therefore, replication is required in studies ideally including non-clinical voice-hearers. The coding framework defined three levels of characterisation with the rationale that the degree of characterisation might be important in the AVATAR therapy context. However, no significant differences were observed between low and medium categories, while effect sizes for differences in behavioural engagement (BAVQ-R) were, unexpectedly, slightly larger for comparisons of high *v.* medium than high *v.* low. Rater disagreements were also limited to the low/medium distinction. Taken together these findings suggest that it may be preferable to dichotomise characterisation into complex (high) *v.* non-complex (low/medium) characterisation consistent with other emerging work (Alderson-Day et al., 2021). Indeed, the secondary analyses adopting this approach showed consistent differences with study hypotheses. The coding framework did not include pre-determined scoring rules and would benefit from refinement to increase standardisation and operationalisation of essential scoring criteria. This work is currently underway and will allow us to report on validity in future studies. Furthermore, while the assessments (and AVATAR therapy) were anchored to the dominant persecutory voice, multiple voices were the norm in this sample and it is possible that certain responses might incorporate aspects of the non-dominant voices which may have affected findings. The association between characterisation and avatar dialogue engagement was less strong for NoE compared to the other measures (although it was significant with a moderate effect size when characterisation was dichotomised). A fine-grained approach, for example the linguistic analysis noted above, would be required to establish the optimal method of measuring interaction in dialogue in future studies.

### Clinical implications

The process of creating an avatar representing the voice (an act of externalisation, personification and embodiment) has been identified as helpful for many, including those with less personified voices (Ward et al., 2020). The AVATAR therapy approach could be framed as changing voice characterisation through dialogue, with the aim of mirrored change in the everyday voice. It remains an open question as to whether AVATAR therapy may be most indicated where complex personification is ‘in play’ pre-therapy. It might also be argued that more highly characterised voices (i.e. those showing complex personification) may be particularly amenable to other approaches which share AVATAR therapy's emphasis on the mirroring of voice relating with other relationships (Hayward et al., 2017) and the understanding of voices within autobiographical context (Corstens et al., 2012; Steel et al., 2019). This might suggest personalising the therapy approach according to the degree and nature of the characterisation. Conversely relational approaches, including AVATAR therapy, may offer equally effective (albeit perhaps differing) therapeutic opportunities for personified and non-personified voices. A new trial is about to start (AVATAR2) which, alongside testing efficacy compared with usual care, will examine optimising and personalising AVATAR therapy (Garety et al. submitted). The efficacy of a brief (six sessions with standardised focus on exposure and assertiveness) and extended (12 sessions incorporating individual formulation-driven treatment targets) form of AVATAR therapy will be tested compared to usual care, with voice characterisation included as a potential moderator of treatment outcome.

## Conclusion

The current study represents an important initial step in connecting voice characterisation with voice engagement (in and out of therapy). We have found that dominant voices showing complex characterisation are associated with increased voice engagement in daily life and that this is mirrored in more extended AVATAR therapy dialogue. Relational approaches such as AVATAR therapy are changing the landscape of psychological therapy for distressing voices. The experience of voice-hearing is diverse and grounded within personal biography and individual meanings. It is hoped that the integration of phenomenological and clinical research may lead to the development of more effective, personalised therapies for people who experience distressing voices.

## Supplementary material

For supplementary material accompanying this paper visit https://doi.org/10.1017/S0033291721000659. click here to view supplementary material

## Data sharing

According to Wellcome Trust's Policy on data, software and materials management and sharing, all data supporting this study will be available in anonymised form from the corresponding author (TW) on reasonable request, subject to review, following the publication of results.

## References

[ref1] Alderson-Day, B., & Fernyhough, C. (2016). Auditory verbal hallucinations: Social, but how? Journal of Consciousness Studies, 23(7–8), 163–194. 29238264 PMC5724750

[ref2] Alderson-Day, B., Woods, A., Moseley, P., Common, S., Deamer, F., Dodgson, G., … Fernyhough, C. (2021). Voice-Hearing and personification: Characterizing social qualities of auditory verbal hallucinations in early psychosis. Schizophrenia Bulletin, 47(1), 228–236. 33484268 10.1093/schbul/sbaa095 PMC7824995

[ref3] Beavan, V. (2011). Towards a definition of “hearing voices”: A phenomenological approach. Psychosis, 3(1), 63–73.

[ref4] Bell, V. (2013). A community of one: Social cognition and auditory verbal hallucinations. PLoS Biology, 11(12), e1001723. 24311984 10.1371/journal.pbio.1001723 PMC3848915

[ref5] Birchwood, M., & Chadwick, P. (1997). The omnipotence of voices: Testing the validity of a cognitive model. Psychological Medicine, 27(6), 1345–1353. doi: 10.1017/s0033291797005552. 9403906

[ref6] Birchwood, M., Gilbert, P., Gilbert, J., Trower, P., Meaden, A., Hay, J., … Miles, J. N. (2004). Interpersonal and role-related schema influence the relationship with the dominant ‘voice’ in schizophrenia: A comparison of three models. Psychological Medicine, 34(8), 1571–1580. doi: 10.1017/s0033291704002636. 15724887

[ref7] Birchwood, M., Meaden, A., Trower, P., Gilbert, P., & Plaistow, J. (2000). The power and omnipotence of voices: Subordination and entrapment by voices and significant others. Psychological Medicine, 30(2), 337–344. doi: 10.1017/s0033291799001828. 10824654

[ref8] Birchwood, M., Michail, M., Meaden, A., Tarrier, N., Lewis, S., Wykes, T., … Peters, E. (2014). Cognitive behaviour therapy to prevent harmful compliance with command hallucinations (COMMAND): A randomised controlled trial. The Lancet Psychiatry, 1(1), 23–33. doi: 10.1016/s2215-0366(14)70247-0. 26360400

[ref9] Birtchnell, J. (1996). How humans relate: A new interpersonal theory. London, UK: Psychology Press.

[ref10] Bogen-Johnston, L., deVisser, R., Strauss, C., & Hayward, M. (2019). “It's just a bit like a rollercoaster”: A longitudinal qualitative study exploring a model of the phases of voice hearing. Psychosis, 11(4), 308–318.

[ref11] Chadwick, P., & Birchwood, M. (1994). The omnipotence of voices. A cognitive approach to auditory hallucinations. British Journal of Psychiatry, 164(2), 190–201. doi: 10.1192/bjp.164.2.190. 8173822

[ref12] Chadwick, P., Lees, S., & Birchwood, M. (2000). The revised beliefs about voices questionnaire (BAVQ-R). British Journal of Psychiatry, 177, 229–232. doi: 10.1192/bjp.177.3.229. 11040883

[ref13] Corstens, D., Longden, E., & May, R. (2012). Talking with voices: Exploring what is expressed by the voices people hear. Psychosis, 4(2), 95–104.

[ref14] Corstens, D., Longden, E., McCarthy-Jones, S., Waddingham, R., & Thomas, N. (2014). Emerging perspectives from the hearing voices movement: Implications for research and practice. Schizophrenia Bulletin 40, S285-S294. 24936088 10.1093/schbul/sbu007 PMC4141309

[ref15] Craig, T. K., Rus-Calafell, M., & Ward, T. (2016). AVATAR therapy for refractory auditory hallucinations. Brief interventions for psychosis (Chapter. 4, pp. 41–54). New York: Springer Nature. 27386617

[ref16] Craig, T. K., Rus-Calafell, M., Ward, T., Leff, J. P., Huckvale, M., Howarth, E., … Garety, P. A. (2018). AVATAR Therapy for auditory verbal hallucinations in people with psychosis: A single-blind, randomised controlled trial. The Lancet Psychiatry, 5(1), 31–40. Retrieved from https://www.thelancet.com/pdfs/journals/lanpsy/PIIS2215-0366(17)30427-3.pdf. 29175276 10.1016/S2215-0366(17)30427-3 PMC5746597

[ref17] David, A. (2004). The cognitive neuropsychiatry of auditory verbal hallucinations: An overview. Cognitive Neuropsychiatry, 9(1–2), 107–123. 16571577 10.1080/13546800344000183

[ref18] Deamer, F., & Wilkinson, S. (2015). The speaker behind the voice: Therapeutic lessons from pragmatic theory. Frontiers in psychology, 6, 1–5. 26124738 10.3389/fpsyg.2015.00817 PMC4463863

[ref19] Garety, P., Edwards, C. J., Ward, T., Emsley, R., Huckvale, M., McCrone, P., … Craig, T. Optimising AVATAR therapy for distressing voices: Study protocol for the AVATAR2 multi-centre randomised controlled trial (submitted to trials). 10.1186/s13063-021-05301-w PMC8145186 34034792

[ref20] Gilbert, P., & Allan, S. (1994). Assertiveness, submissive behaviour and social comparison. British Journal of Clinical Psychology, 33(3), 295–306. 7994215 10.1111/j.2044-8260.1994.tb01125.x

[ref21] Gilbert, P., Birchwood, M., Gilbert, J., Trower, P., Hay, J., Murray, B., … Miles, J. N. (2001). An exploration of evolved mental mechanisms for dominant and subordinate behaviour in relation to auditory hallucinations in schizophrenia and critical thoughts in depression. Psychological Medicine, 31(6), 1117–1127. doi: 10.1017/s0033291701004093. 11513379

[ref22] Haddock, G., McCarron, J., Tarrier, N., & Faragher, E. B. (1999). Scales to measure dimensions of hallucinations and delusions: The psychotic symptom rating scales (PSYRATS). Psychological Medicine, 29(4), 879–889. doi: 10.1017/S0033291799008661. 10473315

[ref23] Hartigan, N., McCarthy-Jones, S., & Hayward, M. (2014). Hear today, not gone tomorrow? An exploratory longitudinal study of auditory verbal hallucinations (hearing voices). Behavioural and Cognitive Psychotherapy, 42(1), 117. 23866079 10.1017/S1352465813000611

[ref24] Hayward, M. (2003). Interpersonal relating and voice hearing: To what extent does relating to the voice reflect social relating? Psychology and Psychotherapy: Theory. Research and Practice, 76(4), 369–383. 10.1348/147608303770584737 14670187

[ref25] Hayward, M., Berry, K., & Ashton, A. (2011). Applying interpersonal theories to the understanding of and therapy for auditory hallucinations: A review of the literature and directions for further research. Clinical Psychology Review, 31(8), 1313–1323. doi: 10.1016/j.cpr.2011.09.001 21996292

[ref26] Hayward, M., Jones, A.-M., Bogen-Johnston, L., Thomas, N., & Strauss, C. (2017). Relating therapy for distressing auditory hallucinations: A pilot randomized controlled trial. Schizophrenia Research, 183, 137–142. 27916286 10.1016/j.schres.2016.11.019

[ref27] Hayward, M., Overton, J., Dorey, T., & Denney, J. (2009). Relating therapy for people who hear voices: A case series. Clinical Psychology & Psychotherapy, 16(3), 216–227. doi: 10.1002/cpp.615 19455717

[ref28] Leff, J., Williams, G., Huckvale, M., Arbuthnot, M., & Leff, A. P. (2014). Avatar therapy for persecutory auditory hallucinations: What is it and how does it work? Psychosis, 6(2), 166–176. 24999369 10.1080/17522439.2013.773457 PMC4066885

[ref29] Leff, J., Williams, G., Huckvale, M. A., Arbuthnot, M., & Leff, A. P. (2013). Computer-assisted therapy for medication-resistant auditory hallucinations: Proof-of-concept study. British Journal of Psychiatry, 202, 428–433. doi: 10.1192/bjp.bp.112.124883. 23429202

[ref30] Longden, E., Corstens, D., Escher, S., & Romme, M. (2012). Voice hearing in a biographical context: A model for formulating the relationship between voices and life history. Psychosis, 4(3), 224–234.

[ref31] Mawson, A., Cohen, K., & Berry, K. (2010). Reviewing evidence for the cognitive model of auditory hallucinations: The relationship between cognitive voice appraisals and distress during psychosis. Clinical Psychology Review, 30(2), 248–258. doi: 10.1016/j.cpr.2009.11.006. 20071062

[ref32] Myin-Germeys, I., Kasanova, Z., Vaessen, T., Vachon, H., Kirtley, O., Viechtbauer, W., & Reininghaus, U. (2018). Experience sampling methodology in mental health research: New insights and technical developments. World Psychiatry, 17(2), 123–132. 29856567 10.1002/wps.20513 PMC5980621

[ref33] Paulik, G. (2012). The role of social schema in the experience of auditory hallucinations: A systematic review and a proposal for the inclusion of social schema in a cognitive behavioural model of voice hearing. Clinical Psychology & Psychotherapy, 19(6), 459–472. doi: 10.1002/cpp.768. 21774037

[ref34] Romme, M., Escher, S., Dillon, J., Corstens, D., & Morris, M. (2009). Living with voices: 50 stories of recovery: PCCS books, Monmouth, UK.

[ref35] Rosenthal, R. (1991). Meta-Analytic procedures for social research (rev. ed.). applied social research methods series, Vol. 6. Thousand Oaks, CA: Sage Publications, Inc; US.

[ref36] Rus-Calafell, M., Ward, T., Zhang, X. C., Edwards, C. J., Garety, P., & Craig, T. (2020). The role of sense of voice presence and anxiety reduction in AVATAR therapy. Journal of Clinical Medicine, 9(9), 2748. 32854387 10.3390/jcm9092748 PMC7564300

[ref37] Steel, C., Schnackenberg, J., Perry, H., Longden, E., Greenfield, E., & Corstens, D. (2019). Making sense of voices: A case series. Psychosis, 11(1), 3–15.

[ref38] Ward, T., Rus-Calafell, M., Ramadhan, Z., Soumelidou, O., Fornells-Ambrojo, M., Garety, P., … Craig, T. K. (2020). AVATAR Therapy for distressing voices: A comprehensive account of therapeutic targets. Schizophrenia Bulletin, ***46***(5), 1038-1044. 10.1093/schbul/sbaa061 PMC7505185 32372082

[ref39] Waters, F., Allen, P., Aleman, A., Fernyhough, C., Woodward, T. S., Badcock, J. C., … Menon, M. (2012). Auditory hallucinations in schizophrenia and nonschizophrenia populations: A review and integrated model of cognitive mechanisms. Schizophrenia Bulletin, 38(4), 683–693. 22446568 10.1093/schbul/sbs045 PMC3406530

[ref40] Wilkinson, S., & Bell, V. (2016). The representation of agents in auditory verbal hallucinations. Mind & Language, 31(1), 104–126. 26900201 10.1111/mila.12096 PMC4744949

[ref41] Woods, A., Jones, N., Alderson-Day, B., Callard, F., & Fernyhough, C. (2015). Experiences of hearing voices: Analysis of a novel phenomenological survey. The Lancet Psychiatry, 2(4), 323–331. Retrieved from https://www.thelancet.com/pdfs/journals/lanpsy/PIIS2215-0366(15)00006-1.pdf. 26360085 10.1016/S2215-0366(15)00006-1 PMC4580735

