# Supplementary material for: The role of characterisation in everyday voice engagement and AVATAR therapy dialogue
Source: Psychol Med. 2021 Apr 8;52(16):3846–53. doi: 10.1017/S0033291721000659 (PMC9811344; doi:10.1017/S0033291721000659)
Supplement: Supplementary file 1 [file S0033291721000659sup001.docx]

| **Supplementary Table 1.** Associations between voice characterisation (rated low/medium/high) and secondary measures of AVATAR dialogue engagement | | | | | |
| --- | --- | --- | --- | --- | --- |
|  | High  (N=18) | Medium  (N=18) | Low  (N=9) | Statistic | Individual comparisons:  Mean difference (95 CI), p (2-tailed), Hedges’ g (95% CI) |
| AVATAR engagement  - total words overall  Mean  (SD) | 2103.28  (807.14) | 1328.22  (580.26) | 1284.67  (455.48) | F=7.67, p=.001, η^2^=.268 | High vs. Medium: 775.06 (224.00 to 1326.11), p=.003, g=1.08 (0.38 to 1.76)  High vs. Low: 818.61 (143.71 to 1493.51), p=.012, g=1.11 (0.27 to 1.94)  Medium vs. Low: 43.56 (-718.45 to 631.34), p=.986, g=0.08 (-0.70 to 0.85) |
| AVATAR engagement  - total words person  Mean  (SD) | 1176.89  (622.30) | 676.61  (513.38) | 641.00  (299.45) | F=5.06, p=.011, η^2^=.194 | High vs. Medium: 500.28 (60.04 to 940.52), p=.019, g=0.86 (0.18 to 1.52)  High vs. Low: 535.89 (-3.29 to 1075.07), p=.045, g=0.96 (0.13 to 1.77)  Medium vs. Low: 35.61 (-503.57 to 574.79 to), p=.985, g=0.08 (-0.70 to 0.85) |
| AVATAR engagement  - Number of Exchanges  Mean  (SD) | 137.44  (60.26) | 95.17  (49.17) | 111.22  (64.32) | F=2.51, p=.093, η^2^=.107 |  |

| **Supplementary Table 2.** Differences in BAVQ-subscales between high (complex) characterisation and medium/low (non-complex) characterisation. | | | |
| --- | --- | --- | --- |
|  | High (complex) (n=20) | Low/Medium (non-complex) (n=40) | z (standardised test score, p,  Effect size r* |
| Behavioural Engagement: Median (IQR) | 3 (3.5) | 0 (3) | z= -2.732, p=.006,  r=0.35 |
| Emotional Engagement: Median (IQR) | 2(5) | 0 (0.5) | z=-2.906, p=.004,  r=0.38 |
| Behavioural Resistance:  Median (IQR) | 10 (7.5) | 10 (4.5) | z= 0.631, p=.528,  r=.08 |
| Emotional Resistance:  Median (IQR) | 8.5 (3.5) | 9 (4) | z= 0.534, p=.593,  r= .07 |
| * For non-parametric tests effect size r=Z/√N where z is the standardised test-score and N= number of observations (Rosenthal, 1991)- For r as an effect size .1= small, .3= medium, .5= large | | | |

| **Supplementary Table 3.** Associations between AVATAR dialogue engagement and characterisation (rated high (complex) characterisation vs. medium/low (non-complex) characterisation. | | | | | |
| --- | --- | --- | --- | --- | --- |
|  | High Mean (SD) n=18 | Low-Medium Mean (SD); n=27 | Mean Difference (95%CI) | Test statistic,  p | Hedges g (95% CI) |
| Total Conversation Time (sec) | 915.11 (303.32) | 627.30 (227.27) | 287.82 (128.26 to 447.37 | t(43)= 3.638, p=.001 | 1.09 (0.45 to 1.71) |
| Total Words (overall) | 2103.28 (807.14) | 1313.70 (533.31) | 789.54 (387.39 to 1191.76) | t(43)= 3.959, p<.001 | 1.18 (0.54 to 1.81) |
| Total words (person) | 1176.89 (622.30) | 664.74 (447.45) | 512.15 (190.83 to 833.46) | t(43)= 3.214, p=.002 | 0.96 (0.34 to 1.58) |
| Number of Exchanges (Person-Avatar) | 137.44 (60.26) | 100.52 (53.97) | 36.93 (2.23 to 71.62) | t(43)= 2.146, p=.038 | 0.64 (0.04 to 1.24) |

References

Rosenthal, R. (1991). *Meta-analytic procedures for social research (rev. ed.). Applied social research methods series, Vol. 6*. Thousand Oaks, CA: Sage Publications, Inc; US.
